# DNA Barcode Analysis of Thrips (Thysanoptera) Diversity in Pakistan Reveals Cryptic Species Complexes

**DOI:** 10.1371/journal.pone.0146014

**Published:** 2016-01-07

**Authors:** Romana Iftikhar, Muhammad Ashfaq, Akhtar Rasool, Paul D. N. Hebert

**Affiliations:** 1 National Institute for Biotechnology and Genetic Engineering (NIBGE), Faisalabad, Pakistan; 2 Centre for Biodiversity Genomics, Biodiversity Institute of Ontario, University of Guelph, Guelph, Ontario, Canada; CSIRO, AUSTRALIA

## Abstract

Although thrips are globally important crop pests and vectors of viral disease, species identifications are difficult because of their small size and inconspicuous morphological differences. Sequence variation in the mitochondrial COI-5ʹ (DNA barcode) region has proven effective for the identification of species in many groups of insect pests. We analyzed barcode sequence variation among 471 thrips from various plant hosts in north-central Pakistan. The Barcode Index Number (BIN) system assigned these sequences to 55 BINs, while the Automatic Barcode Gap Discovery detected 56 partitions, a count that coincided with the number of monophyletic lineages recognized by Neighbor-Joining analysis and Bayesian inference. Congeneric species showed an average of 19% sequence divergence (range = 5.6% - 27%) at COI, while intraspecific distances averaged 0.6% (range = 0.0% - 7.6%). BIN analysis suggested that all intraspecific divergence >3.0% actually involved a species complex. In fact, sequences for three major pest species (*Haplothrips reuteri*, *Thrips palmi*, *Thrips tabaci*), and one predatory thrips (*Aeolothrips intermedius*) showed deep intraspecific divergences, providing evidence that each is a cryptic species complex. The study compiles the first barcode reference library for the thrips of Pakistan, and examines global haplotype diversity in four important pest thrips.

## Introduction

Thrips (Thysanoptera) are serious pests and disease vectors on many economically important crops throughout the world [1,2]. Identification of most thrips to a species level is difficult because of their small size, subtle morphological differentiation [3], intraspecific polymorphisms [4], and sexual dimorphisms [5]. Molecular identification of thrips has major advantages to morphology-based analysis because it overcomes the complexities introduced by morphological variation among life stages and the inconspicuous morphological differences among species [3,6]. Several gene regions have been employed for species discrimination [7, 8] and phylogenetic analysis [9]. Crespi et al. [10] employed the nuclear 18S and mitochondrial gene cytochrome *c* oxidase I (COI) genes to examine phylogenetic relationships between two suborders Terebrantia and Tubulifera, while Buckman et al. [9] coupled four nuclear loci (18S & 28S ribosomal DNA (rDNA), Histone 3, Tubulin-alpha 1) with COI to ascertain the phylogenetic relationships of 99 species of thrips from seven families. COI has also been recognized as a particularly suitable marker for thrips identification because it exhibits more consistent interspecific variation [11] than other markers [12,13]. Its analysis has, for example, helped to reveal the number of thrips species inhabiting particular cropping systems [14]. The capacity of the COI-5ʹ (DNA barcode) region to discriminate cryptic species of insects has been well validated [15–18] including for thrips [3,12].

Levels of COI sequence divergence often are helpful in deciding if two sequences derive from different species [19] as most conspecifics show <2% divergence in the barcode region [15,20]. The Barcode Index Number (BIN) system now provides an interim taxonomic system based on COI sequence clusters [21] for all animals and most BINs are congruent with morphological species [22,23]. Barcode data has been used to advance species-level taxonomy in various animal groups [24], often revealing new species [25,26]. Researchers have also employed DNA barcoding to identify pest thrips for quarantine [27,28].

Some pest thrips species are thought to be a complex of multiple cryptic species [29]. For example, COI analysis revealed three lineages of *Thrips tabaci* [6,30], while *Thrips palmi* has two clades [27,13]. Likewise, western flower thrips, *Frankliniella occidentalis*, is now viewed as two species [29]. Members of species complexes have been discriminated by sequence matches [31] or by PCR analysis with species-specific primers, enabling a non-specialist to identify the target species at any life stage [12].

The rapid increase in global trade warrants the development of a universal, anticipatory system with the capacity to identify taxa that are newly encountered in a region because invasive species can reduce local biodiversity and often cause serious economic damage to crops [32]. The accurate identification of pest and invasive species is critical for both control [33,34] and quarantine [35] as misidentifications may lead to ineffective control measures [33]. The varying capacity of thrips species to transmit viral diseases [36] provides an additional incentive to explore their genetic diversity. Prior taxonomic studies on the Thysanoptera of Pakistan [37–43] have been limited in scope, but 76 species have been reported including members of three families (Aeolothripidae, Phlaeothripidae, Thripidae) (Appendix 1). The present study analyzes patterns of COI sequence diversity among thrips from Pakistan, initiating the development of a regional barcode reference library for Thysanoptera. Furthermore, the study examines the broader geographic patterning of haplotype frequencies in four particularly important pest species.

## Materials and Methods

### Ethics Statement

No permits are required to collect thrips, but permission from the landowners was obtained whenever necessary. All the collection sites were in unprotected areas accessible to the public. The study did not involve endangered or protected species.

### Specimen collections

**Fig 1:**
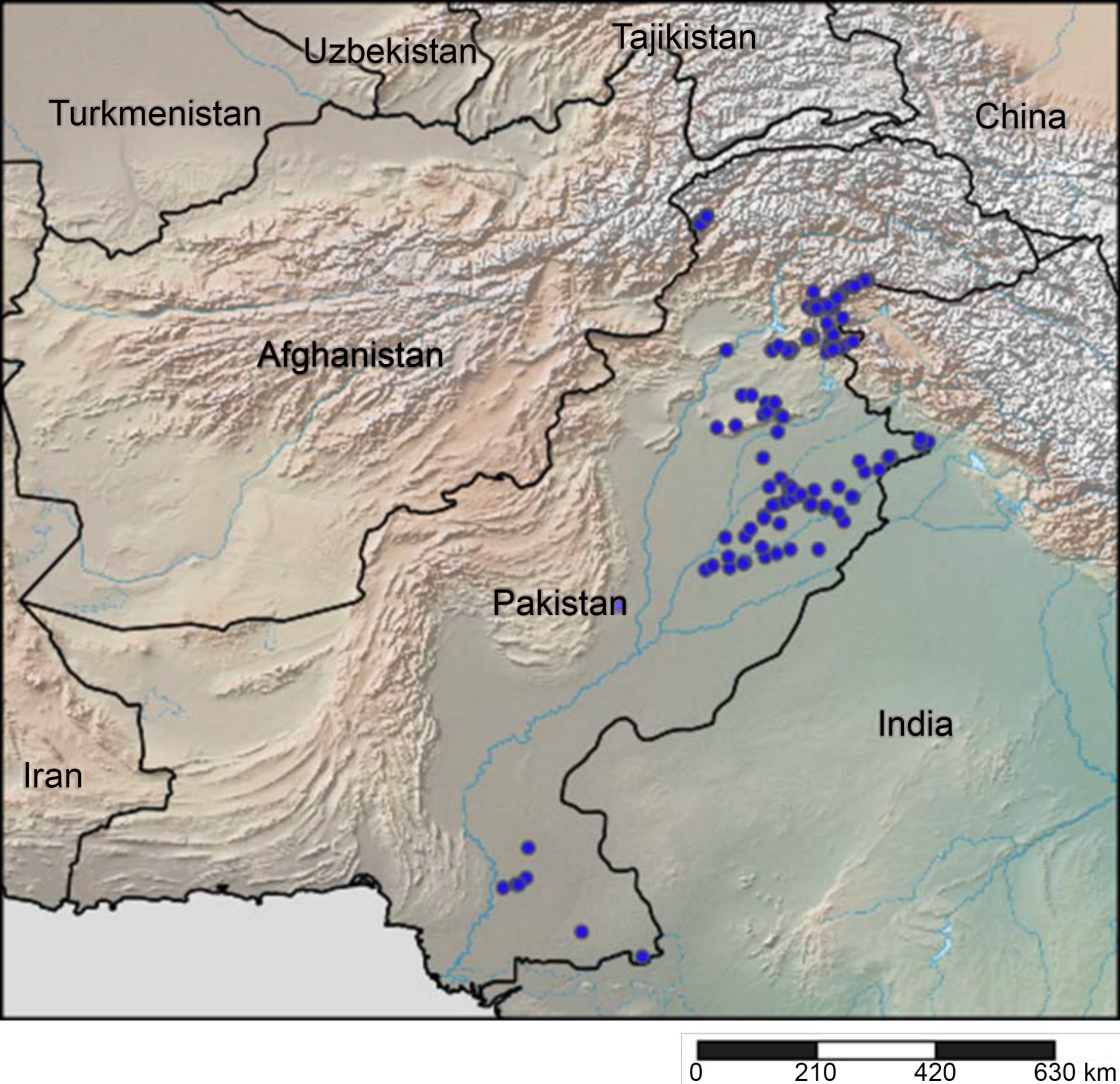
Collection sites for thrips in Pakistan. The map was generated by www.simplemappr.net using GPS coordinates.

Thrips were collected from 158 localities in Pakistan (Fig 1) during 2009–2012 from shrubs, trees, crops, weeds and ornamentals following standard protocols [44]. Thrips were dislodged from plant foliage or inflorescences by beating vegetation over white paper sheets and transferring specimens with a fine camel hair brush to 1.5 ml Eppendorf tubes containing 95% ethanol. The name of the collector, date of collection, and locations with the GPS coordinates were recorded. 495 specimens were collected and stored at -20°C until analysis.

### Databasing

Specimen data were submitted to the Barcode of Life Data Systems (BOLD) (http://www.boldsystems.org) [45] under the project, MATHR "Thrips Species of Pakistan". Prior to DNA extraction, each specimen was photographed and its image was uploaded to BOLD.

### DNA extraction

Thrips were transferred individually, one specimen per well, to 96-well plates in accordance with the specimen data and images submitted to BOLD. DNA isolation was carried out at the Canadian Centre for DNA Barcoding (CCDB) within the Centre for Biodiversity Genomics following protocols described by Porco et al. [46].

### DNA polymerase chain reaction (PCR)

Amplification of the 658 bp COI-5ʹ barcode region was performed with the primers C_LepFolF and C_LepFolR (http://www.ccdb.ca/docs/CCDB_PrimerSets.pdf) following PCR conditions; 94°C (1 min), 5 cycles of 94°C (40 s), 45°C (40 s), 72°C (1 min); 35 cycles of 94°C (40 s), 51°C (40 s), 72°C (1 min) and final extension of 72°C (5 min). These primers are mixtures of LepF1 (ATTCAACCAATCATAAAGATATTGG)/LCO1490 (GGTCAACAAATCATAAAGATATTGG) and LepR1 (TAAACTTCTGGATGTCCAAAAAATCA)/HCO2198 (TAAACTTCAGGGTGACCAAAAAATCA) [47], respectively. Amplification of a 439 bp segment of COI-3ʹ was performed with primer set C1-J-1751 (GGATCACCTGATATAGCATTYCC)/C1-N-2191 (CCCGGTAAAATTAAAATATAAACTTC) under the PCR conditions outlined above. The internal transcribed spacer 1 (ITS1) of the rDNA was amplified using primers CAS18sF1 (TACACACCGCCCGTCGCTACTA) and CAS5p8sB1d (ATGTGCGTTCRAAATGTCGATGTTCA) [48]. Amplifications involved 12.5 μL reactions containing standard PCR ingredients [49] and 2 μL of DNA template. PCR products were analyzed on 2% agarose E-gel® 96 system (Invitrogen Inc.). Amplicons were sequenced bidirectionally using the BigDye Terminator Cycle Sequencing Kit (v3.1) (Applied Biosystems) on an Applied Biosystems 3730XL DNA Analyzer. The forward and reverse sequences were assembled, aligned and edited using CodonCode Aligner (CodonCode Corporation, USA) and submitted to BOLD. Sequences were also inspected and translated in MEGA5 [50] to verify that they were free of stop codons. All sequences generated in this study were submitted to GenBank (S1 Table) and are accessible on BOLD under the dataset DS-MATHR (dx.doi.org/10.5883/DS-MATHR).

### Morphological identification

Specimens were retrieved after DNA extraction [46] and mounted onto slides using Hoyer’s medium. The mounted specimens were identified using descriptions at http://www.ozthrips.org, http://keys.lucidcentral.org/keys/v3/thrips_of_california and standard morphological keys [37–39,51]. Morphological characters were examined using a compound microscope (Olympus BX 41) at 40X, 100X and 400X. Identifications were verified by Stan Diffie (Department of Entomology, University of Georgia, USA) and Sueo Nakahara (USDA ARS, Beltsville, MD, USA). Species names were validated at ThripsWiki (http://thrips.info/wiki/) (accessed on 26 Apr 2014). Voucher specimens were deposited at the Insect Museum National Institute for Biotechnology and Genetic Engineering, Faisalabad, Pakistan.

### Data analysis

All sequences obtained in this study were compared with those on GenBank and BOLD using "BLASTn" (http://www.ncbi.nlm.nih.gov/blast/) or "Identification Request", respectively. BOLD assigns all barcode sequences with lengths >500bp to a BIN so all thrips sequences from this study were assigned to a BIN.

ClustalW nucleotide sequence alignments [52] and Neighbor Joining (NJ) clustering analysis were performed using MEGA5. NJ trees employed the Kimura-2-Parameter (K2P) distance model [53] with pairwise deletion of missing sites and nodal support was estimated using 500 bootstrap replicates. Distance histograms were generated using the online version of Automatic Barcode Gap Discovery (ABGD) [54]. The ‘Barcode Gap Analysis’ (BGA) was performed using tools available on BOLD. The presence or absence of a ‘barcode gap’ was determined for each species to ascertain the reliability of its discrimination [55]. Using the barcode gap criterion, a species is distinct from its nearest neighbor (NN) if its maximum intraspecific distance is less than the distance to its NN sequence [55].

Unique haplotype sequences were extracted from the alignment with DnaSP 5.10 [56]. Phylogenetic trees were constructed from unique haplotype sequences using MrBayes v3.2.0 [57] and the Markov Chain Monte Carlo (MCMC) technique. *Rhopalosiphum padi* (HQ979401) was included as outgroup. The data was partitioned in two ways; i) a single partition with parameters estimated across all codon positions, ii) a codon-partition in which each codon position was allowed different parameter estimates. The analyses were run for 10 million generations using four chains with sampling every 1000 generations. The evolution of sequences was modelled by the GTR+Γ model independently for the two partitions using the ‘‘unlink” command in MrBayes. The model selection was made using FindModel (www.hiv.lanl.gov/cgi-bin/findmodel/findmodel.cgi). Bayesian posterior probabilities were calculated from the sample points once the MCMC algorithm converged. Convergence was determined when the standard deviation of split frequencies was less than 0.002 and the PSRF (potential scale reduction factor) approached 1, and both runs converged to a stationary distribution after the burn-in stage (by default, the first 25% of samples were discarded). Each run produced 100001 samples of which 75001 samples were included. The trees generated through this process were visualized using FigTree v1.4.0.

### Haplotype and distribution analysis

Barcode sequences from Pakistan for four species (*Scirtothrips dorsalis*, *T*. *flavidulus*, *T*. *palmi*, *T*. *tabaci*), which are important pests and virus vectors, were combined with records from other countries and aligned in MEGA5. For this analysis each morphological species was treated as one taxon regardless of the number of lineages/BINs among its barcode sequences. GenBank and BOLD sequences with clearly incorrect species assignments or potential contaminants (returning unexpected alignments or distances) were removed from the analysis. Sequence alignment (fasta file), for each species, was imported into DnaSP 5.10 to reconstruct haplotypes and generate a haplotype data file (nexus). The nexus file was edited to add haplotype-country links and a minimum spanning network (MSN) [58] was constructed in PopART (http://popart.otago.ac.nz) to examine the relationships among haplotypes for each thrips species from different locations. The MSN is based on a minimum spanning tree where a set of sequence types connects all given types without creating any cycles or inferring additional (ancestral) nodes, such that the total length (i.e., the sum of distances between linked sequence types) is minimal, allowing construction of the union of all minimum spanning trees [58].

## Results

### Morphological identification

Morphological identifications assigned 461 specimens to 43 species, and 14 others to five genera, including two *Thrips*, two *Aeolothrips* and one *Haplothrips*. The remaining 20 specimens could only be identified to a family; seven were members of the Phlaeothripidae while 13 were Thripidae. Morphology successfully identified the four major pests and disease vectors (*Thrips flavidulus*, *T*. *palmi*, *T*. *tabaci*, *Scirtothrips dorsalis*) in the region.

### DNA barcode analysis of thrips species

**Fig 2:**
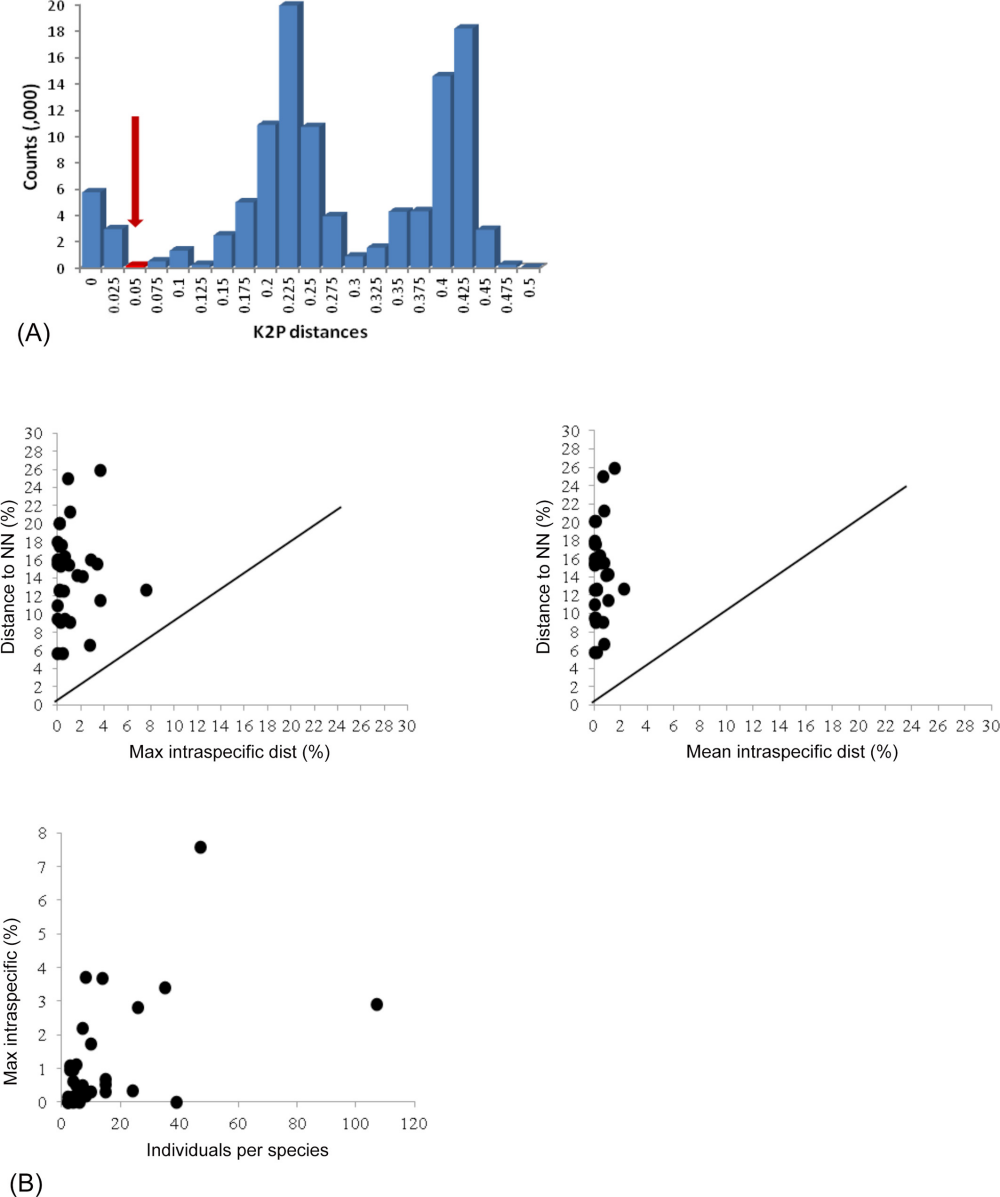
Distribution of pairwise (K2P) distances (A) and barcode gap analysis (B) of thrips from Pakistan. The gap between intraspecific and interspecific distances is indicated by an arrow. Three peaks in the distance bars reflect the sequence divergence among the three families of thrips represented in the dataset. NN = nearest neighbour.

Barcode sequences greater than 500bp were recovered from 471 of the 495 specimens (95%), providing at least one sequence for 42 of the 43 identified species with only *Megalurothrips distalis* lacking coverage. Comparison of the sequences with those in GenBank and BOLD revealed close sequence matches (>98% nucleotide identify) for only 13 of these 42 species. Table 1 shows the sequence divergences among 1) the 31 species with >2 specimens, 2) the five genera with two or more species and 3) the two families with two or more genera. Intra- and interspecific distances averaged 0.6% and 19%, respectively (ranges = 0–7.6% and 5.6–27.0%) (Table 1). Intraspecific distances could not be determined for the 16 species with a single representative, but Nearest-Neighbor (NN) distances for all morphological species were more than 5%. This pattern supported the presence of a barcode gap (the maximum intraspecific distances were less than the distances to the NN sequences). Sequence divergence increased with the taxonomic rank with overlap (2%) between conspecific and congeneric distances detected in only one morphological species, *T*. *palmi*. However, the distance between *T*. *palmi* and its NN (*Thrips florum*) was 12.62%. Distances between the genera in each family averaged 22.3% (range = 6.6–35.6%). Distance (K2P) histograms are presented in Fig 2A. These analyses revealed a gap between maximum intraspecific and minimum interspecific distances in barcodes from the morphological species, supporting the existence of a “barcode gap” (Fig 2A). The ABGD analysis showed 55 initial and 56 recursive partitions at a prior maximal distance of 0.0215. The barcode gap analysis showed that the maximum and mean intraspecific distances for all the species were lower than the distances to their nearest-neighbors (Fig 2B). Maximum intraspecific distances were less than 2% in all species excepting *Aeolothrips intermedius* (3.7%), *Haplothrips reuteri* (3.7%), *T*. *palmi* (7.6%) and *T*. *tabaci* (3.7%).

**Table 1:** Percentage sequence divergence (K2P) at the COI barcode region for the 31 thrips species with two or more specimens, the five genera with two or more species and the two families with two or more genera.

Taxonomic Level	n	Taxa	Comparisons	Min (%)	Mean (%)	Max (%)
Species	439	31	9119	0.0	0.6	7.6
Genus	363	5	22313	5.6	19.0	27.0
Family	438	2	28990	6.6	22.3	35.6

The 471 sequences were assigned to 55 BINs. Morphological identifications and BINs were congruent for 39 species, while sequences for the other three species (*A*. *intermedius*, *H*. *reuteri*, *T*. *palmi*) were split into two BINs. The remaining BINs represented 10 unidentified lineages. Maximum intra-BIN distances were <2% for all 39 species assigned to one BIN except for *T*. *flavidulus* (2.7%) and *T*. *tabaci* (3.7%). The BINs and maximum intraspecific distances for thrips species from Pakistan and other countries are shown in Table 2.

**Table 2:** Barcode Index Numbers (BINs) and maximum intraspecific distances for thrips species collected from Pakistan and barcode coverage from other countries.

			Max intraspecific distance % (records)		
Family	Species	BIN	Pakistan	Combined	Countries with matches
Aeolothripidae	*Aeolothrips intermedius*	AAU0572 AAZ8618	3.7 (13)	NA	NA
	*Aeolothrips PK01*	AAN6626	*	NA	NA
	*Aeolothrips PK02*	AAZ8619	0.2 (3)	NA	NA
	*Ananthakrishnana euphorbiae*	ACA2783	*	NA	NA
Phlaeothripidae	*Apterygothrips pellucidus*	AAY6328	0.0 (2)	NA	NA
	*Haplothrips andresi*	AAN5799	0.2 (6)	NA	NA
	*Haplothrips bagrolis*	AAZ8515	*	NA	NA
	*Haplothrips ciliatus*	AAU5460	0.5 (5)	NA	NA
	*Haplothrips ganglbaueri*	ACF1370	0.0 (39)	NA	NA
	*Haplothrips gowdeyi*	AAN5798	*	NA	NA
	*Haplothrips reuteri*	ACA2784 AAI6863	3.7 (8)	NA	NA
	*Haplothrips PK01*	ACA2828	*	NA	NA
	*Haplothrips stylatus*	AAU6351	*	NA	NA
	*Haplothrips tenuipennis*	AAN4488	2.3 (26)	NA	NA
	*Liothrips infrequens*	ACA2829	*	NA	NA
	*Plicothrips apicalis*	AAN6622	0.3 (15)	NA	NA
	Phlaeothripidae1	ACK3864	0.2 (6)	NA	NA
	Phlaeothripidae2	ACA9557	*	NA	NA
	*Anaphothrips sudanensis*	AAV3388	*	NA	NA
	*Arorathrips mexicanus*	AAN5064	0.2 (4)	NA	NA
Thripidae	*Chaetenaphothrips orchidii*	AAP7685	0.6 (4)	NA	NA
	*Chirothrips meridionalis*	AAN5797	0.0 (5)	0.8 (9)	Croatia
	*Dendrothripoides innoxius*	AAN5065	0.2 (8)	NA	NA
	*Frankliniella schultzei*	AAN6620	0.5 (25)	0.5 (31)	Australia, India, Kenya
	*Hydatothrips atactus*	AAN9110	*	NA	NA
	*Lefroyothrips lefroyi*	ACI6048	*	NA	China, India
	*Megalurothrips peculiaris*	AAN6623	0.3 (10)	0.7 (20)	China
	*Megalurothrips usitatus*	AAM8053	1.1 (5)	1.7 (11)	Australia, India
	*Microcephalothrips abdominalis*	AAI0410	0.3 (15)	1.4 (27)	Australia, USA, Canada, China
	*Mycterothrips nilgiriensis*	ACA2806	*	NA	NA
	*Neohydatothrips samayunkur*	AAP7680	0.2 (7)	0.2 (10)	South Africa
	*Pseudodendrothrips bhattii*	ACG8261	*	NA	NA
	*Scirtothrips dorsalis*	AAC9747 AAC9749 AAC9750 AAC9751 ACQ0434 ACQ0435 ACQ4218 ACV6509 ACV6510 ACV6511 ACV7644	1.7 (10)	20.8 (249)	Australia, Cambodia, China, India, Israel, Japan, Kenya, Singapore, South Korea, Taiwan, Thailand, USA, Vietnam
	*Scirtothrips oligochaetus*	AAZ8518	*	NA	NA
	*Scolothrips rhagebianus*	AAZ8517	*	NA	NA
	*Taeniothrips major*	AAN6621	0.2 (4)	NA	NA
	*Thrips alatus*	AAN6625	0.0 (2)	NA	NA
	*Thrips apicatus*	AAY6262	0.2 (5)	NA	NA
	*Thrips carthami*	AAP7682	0.5 (7)	NA	NA
	*Thrips coloratus*	AAK1804	0.5 (15)	NA	NA
	*Thrips decens*	AAP7679	*	NA	NA
	*Thrips flavidulus*	AAN6624	2.7 (106)	2.7 (114)	China
	*Thrips florum*	AAP7683	0.2 (2)	NA	NA
	*Thrips hawaiiensis*	AAZ8516	0.0 (2)	1.3 (10)	China, India
	*Thrips palmi*	AAE7913 AAN2747	7.6 (8)	13.0 (212)	China, Dominican-Republic India, Japan, Singapore, Switzerland, Taiwan, Thailand, UK, USA
	*Thrips tabaci*	AAB3870	3.7 (36)	12.0 (282)	Australia, Bosnia-Herzegovina, Canada, China, Germany, Greece, India, Iran, Israel, Japan, Kenya, Lithuania, Madagascar, Netherlands, New Zealand, Peru, Serbia, South Africa, Switzerland, Tanzania, UK, USA
	*Thrips trehernei*	AAN9105	0.0 (4)	2.4 (106)	Canada, China, Croatia, Germany, Iran, UK
	*Thrips PK01*	AAN9111	2.2 (7)	NA	NA
	*Thrips PK02*	AAP7684	*	NA	NA
	Thripidae1	ACA3048	0.0 (3)	NA	NA
	Thripidae2	AAP7681	0.4 (5)	NA	NA
	Thripidae3	ACP4916	*	NA	NA

* Species with a single record.

NA = match not available.

**Fig 3:**
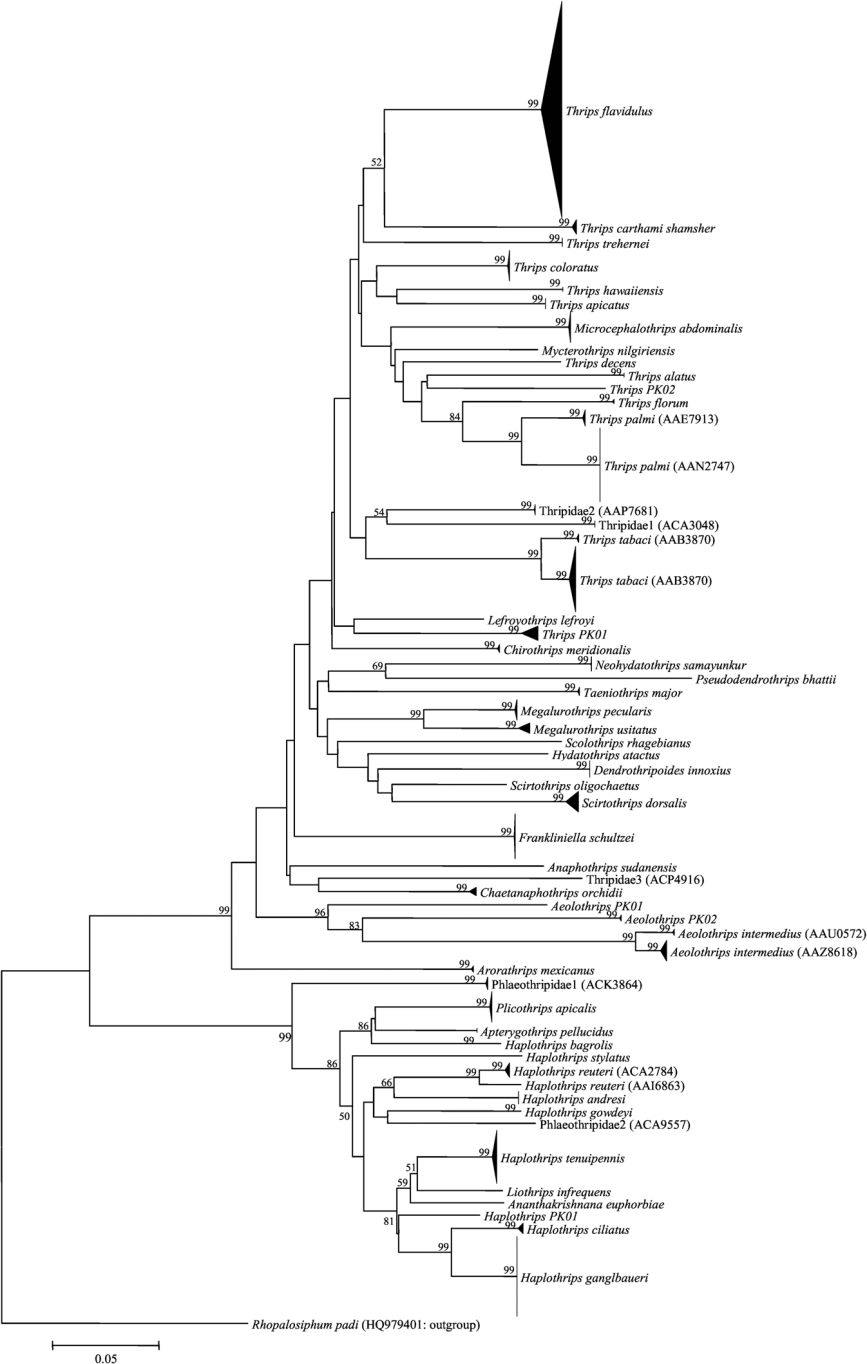
NJ analysis of COI-5′ sequences from species of thrips from Pakistan. Bootstrap values (%) (500 replicates) are shown above the branches (values <50% are not shown) while the scale bar shows K2P distances. The node for each species with multiple specimens was collapsed to a vertical line or triangle, with the horizontal depth indicating the level of intraspecific divergence. BIN numbers are shown for species with only family-level identification or those split into two BINs.

**Fig 4:**
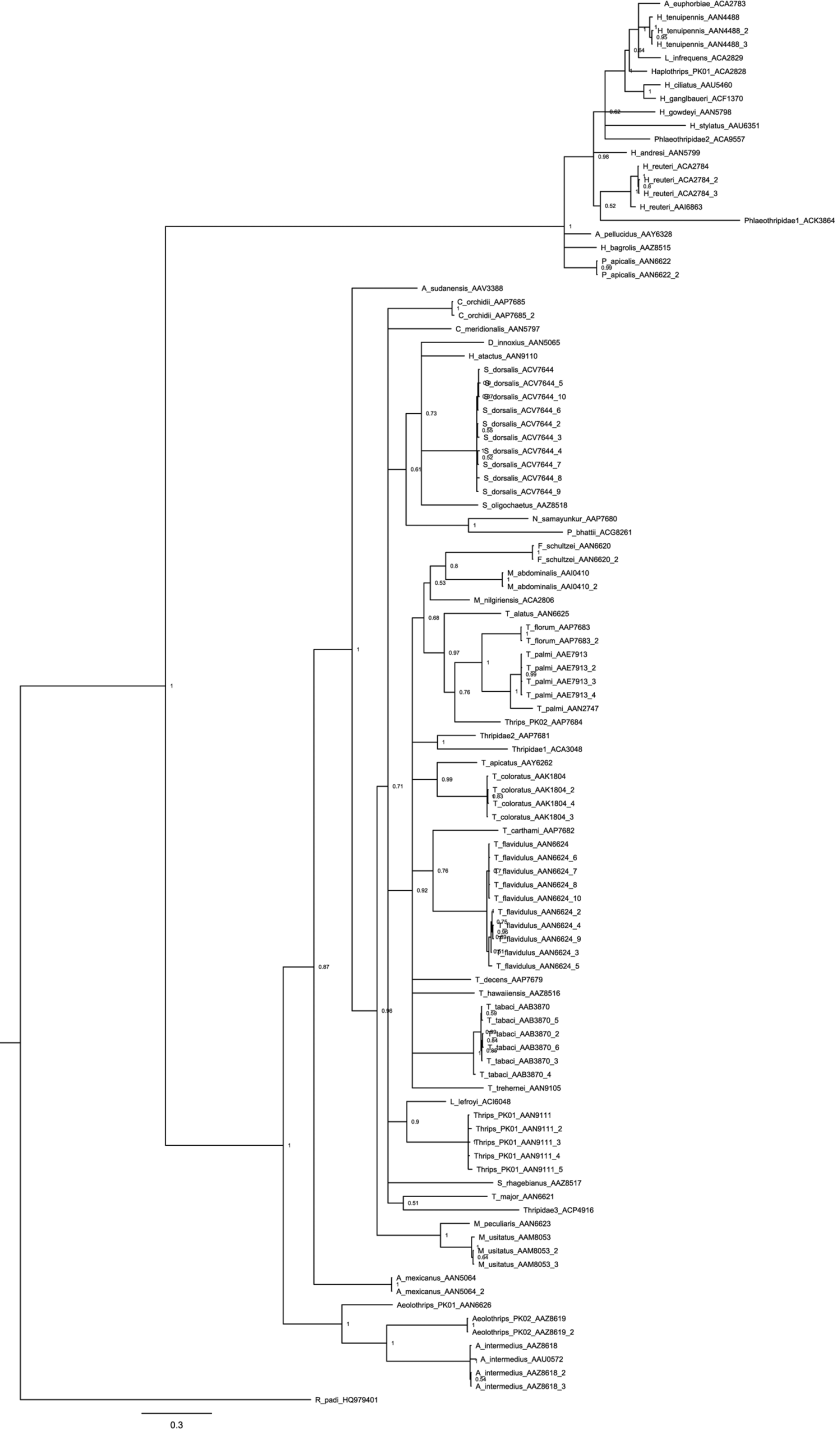
Phylogenetic analysis of thrips species from Pakistan based on COI-5ʹ sequences. The tree was estimated using Bayesian inference. Posterior probabilities are indicated at nodes. Taxa are followed by the BINs and haplotype numbers. *Rhopalosiphum padi* (HQ979401) was employed as outgroup.

**Fig 5:**
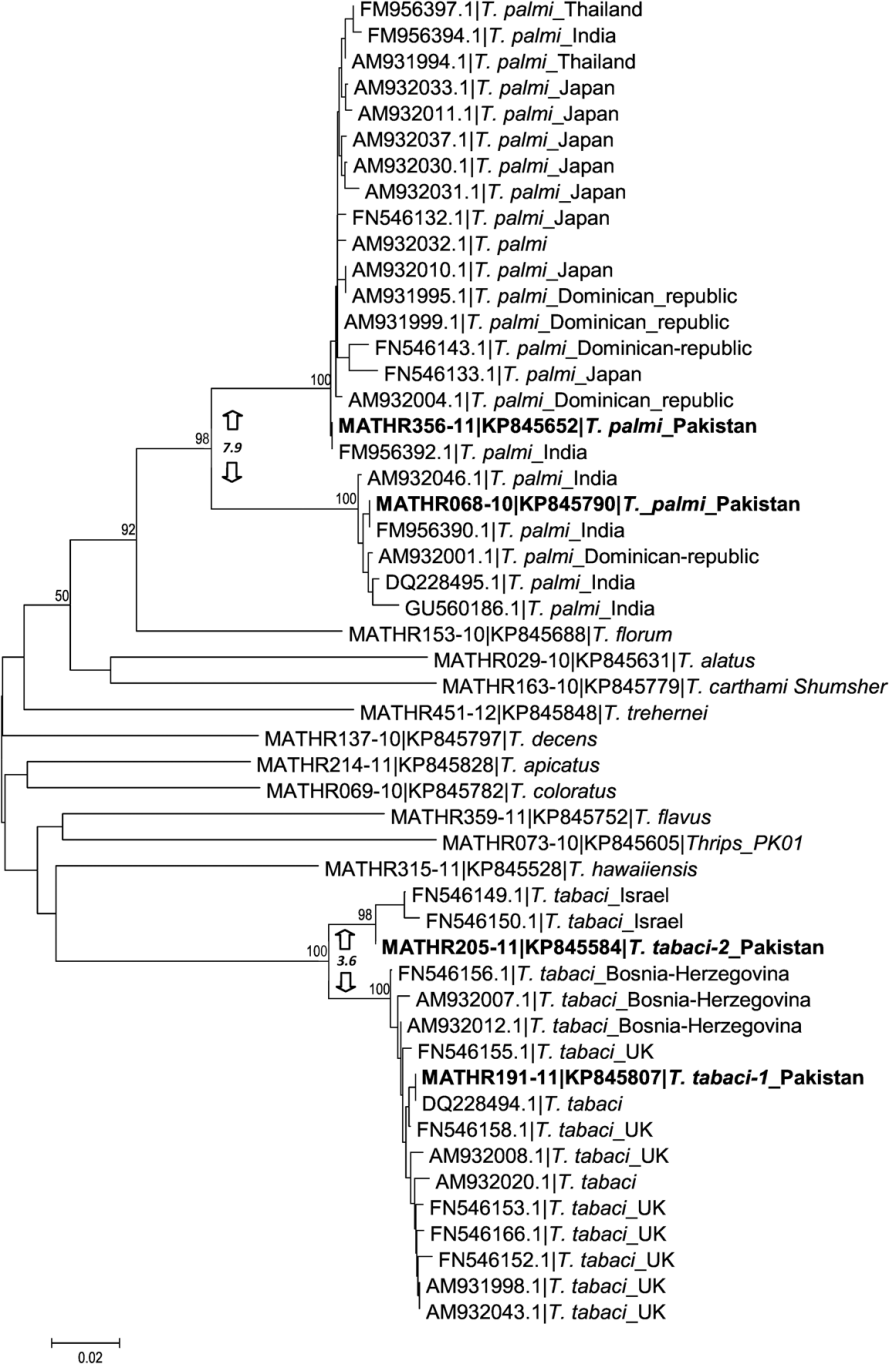
NJ analysis based on COI-3′ sequences from *Thrips palmi* and *Thrips tabaci* from Pakistan (bold letters) with their closest matches (>99% nucleotide identity) from GenBank and other species of *Thrips* in this study. The country of collection follows the species names. K2P divergence within *T*. *palmi* and *T*. *tabaci* clusters is indicated between arrows.

NJ analysis, which included all 471 sequences, showed that sequences from each of the 55 BINs formed a monophyletic clade (Fig 3). Although all specimens of *T*. *tabaci* were assigned to one BIN, the NJ analysis showed two distinct clusters with a mean divergence of 3.4% and Bayesian analysis confirmed the reciprocal monophyly of these two clusters (Fig 4). As both un-partitioned and codon-partitioned trees produced similar topology, only the un-partitioned tree is presented (Fig 4). No prior study has examined sequence variation in the barcode region for the two most important species (*T*. *palmi*, *T*. *tabaci*) of pest Thysanoptera in Pakistan, impeding analysis of the broader geographical distribution of the two COI-5ʹ lineages in these two species. Because more data was available for COI-3ʹ, we sequenced this gene region from both lineages of these two species. NJ analysis of COI-3ʹ from *T*. *palmi* revealed two divergent (min K2P = 7.9% versus 6.4% at COI-5ʹ) lineages; one (BIN: AAE7913) matched (>99% nucleotide identity) specimens from the Dominican Republic, India, Japan, and Thailand, while the other (BIN: AAN2747) matched records from the Dominican Republic and India (Fig 5). Analysis of the COI-3ʹ from *T*. *tabaci* also showed two lineages (min K2P = 3.6%); one matched a lineage from the UK and Bosnia-Herzegovina, while the other matched specimens from Israel (Fig 5).

ITS1 was sequenced from the representative BINs of *A*. *intermedius*, *H*. *reuteri* and *T*. *palmi* and the two barcode lineages of *T*. *tabaci* to determine if sequence variation in this nuclear gene region supported the presence of cryptic species. This analysis revealed substantial length variation in ITS1 among the four species—*A*. *intermedius* 889 bp, *H*. *reuteri* 513 bp, *T*. *palmi* 840 bp and *T*. *tabaci* 965 bp. The minimum K2P divergence (pairwise deletions) between the BINs of *A*. *intermedius* (AAU0552 vs. AAZ8618), *H*. *reuteri* (AAI6863 vs. ACA2784) and *T*. *palmi* (AAE7913 vs. AAN2747) was 1.4%, 4.3% and 2.3% respectively, while divergence between the two barcode lineages of *T*. *tabaci* was 1.5%.

### Global haplotype diversity

**Fig 6:**
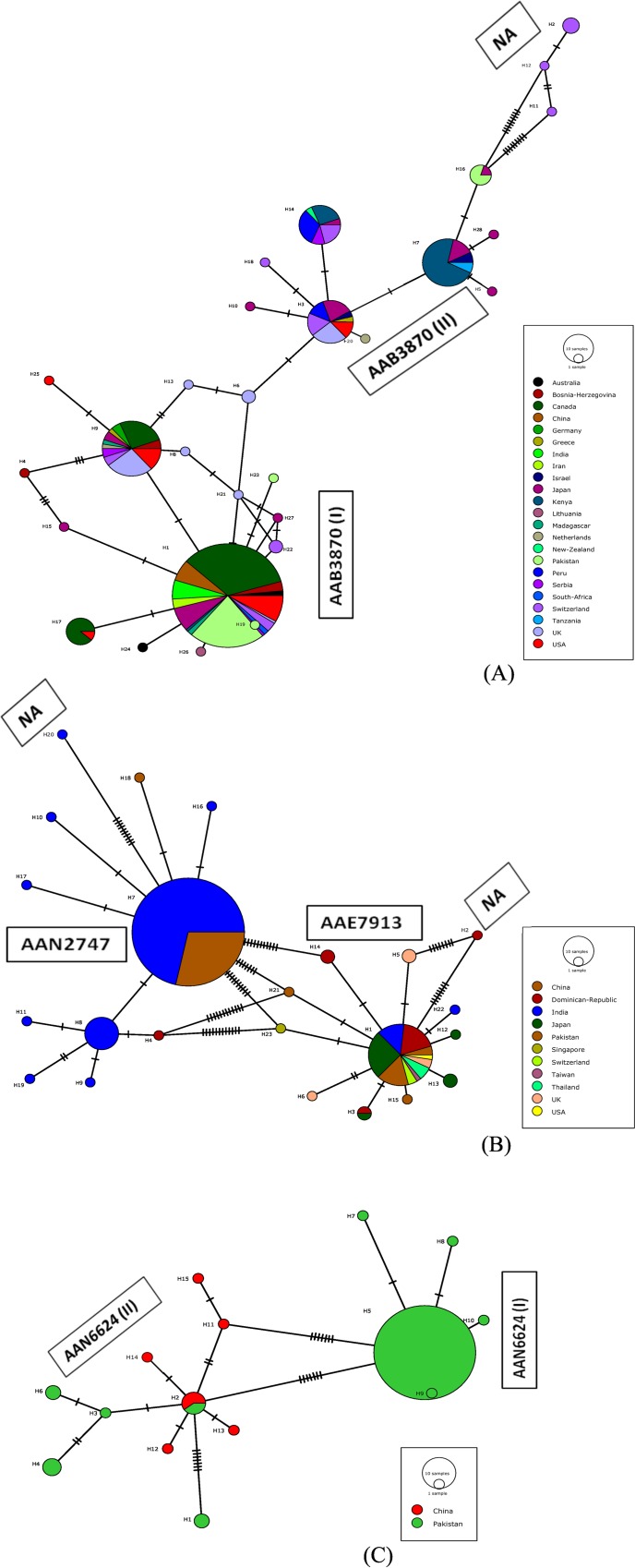
Haplotype networks for three species of pest thrips from Pakistan based on COI-5ʹ sequences including presumptive conspecifics from GenBank. A) *Thrips tabaci;* B) *Thrips palmi;* C) *Thrips flavidulus*. Different pie colors in the circles indicate the country of haplotype origin with pie size proportional to the number of records, while the circle sizes are proportional to the haplotype frequency in the dataset. Perpendicular tick marks on the lines represent the number of nucleotide substitutions between the linked haplotypes. Corresponding BINs are provided besides each haplotype cluster. NA = BIN not assigned.

**Fig 7:**
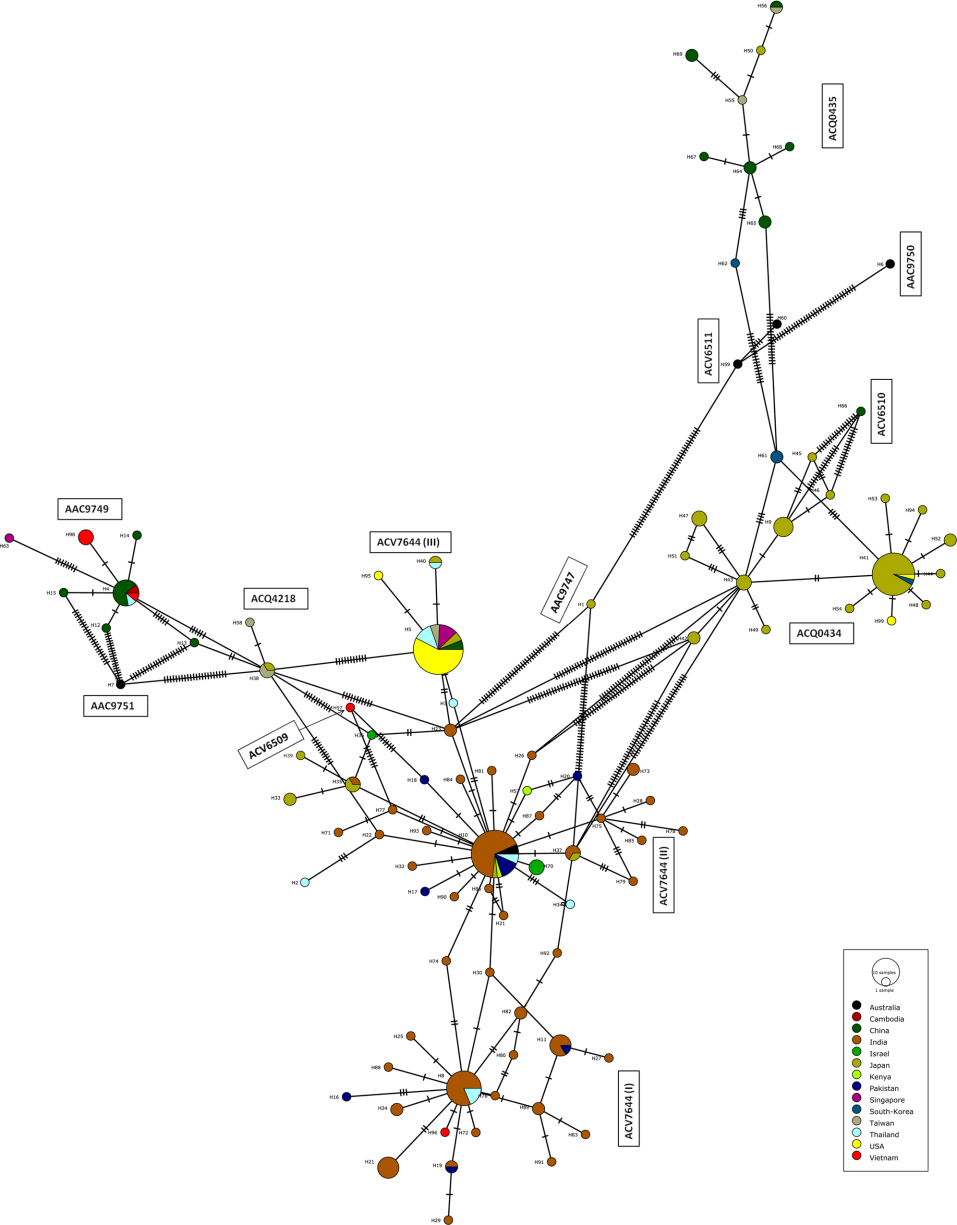
Haplotype network for *Scirtothrips dorsalis* from Pakistan based on COI-5ʹ sequences including conspecifics from GenBank. Different pie colors in the circles indicate the country of haplotype origin with pie size proportional to the number of records, while the circle sizes are proportional to the haplotype frequency in the dataset. Perpendicular tick marks on the lines represent the number of nucleotide substitutions between the linked haplotypes. Corresponding BINs are provided besides each haplotype cluster.

COI-5ʹ sequences from four pest thrips (*T*. *tabaci*, *T*. *palmi*, *T*. *flavidulus*, *S*. *dorsalis*) were combined with those from GenBank to construct haplotype networks (Figs 6 and 7). Analysis focused on these four taxa because too few records were available for the other species to generate meaningful conclusions. The 36 sequences of *T*. *tabaci* from Pakistan combined with 246 from GenBank from four geographic regions (Asia, Europe, Australia and America; 23 countries) revealed 28 haplotypes which clustered into three groups connected by the haplotype from the United Kingdom (Fig 6A). The commonest haplotype (*n* = 135) occurred in 15 countries including Pakistan, while three low frequency haplotypes were only found in Switzerland [6] and comprised a single cluster in the network. In a similar fashion, 47 sequences of *T*. *palmi* from Pakistan were combined with 165 from GenBank (11 countries) revealing 23 haplotypes, one known from all the countries, except for Singapore (Fig 6B). The most common haplotype (*n* = 133) was shared between India and Pakistan. A majority of the haplotypes were clustered into two groups with records from Pakistan found in both groups. However, one haplotype from the Dominican Republic [13] and one from India [14], with eight and ten nucleotide substitutions from their respective NNs, represented singleton clusters (Fig 6B). There were five haplotypes of *T*. *palmi* in Pakistan and two were only found in this country. The 114 records for *T*. *flavidulus* (106 from Pakistan) included 15 haplotypes (Fig 6C) in two clusters. Thirteen were only represented by 1–3 records, most detected only in Pakistan. The dominant haplotype (*n* = 92) was in Pakistan. Among the six haplotypes of *T*. *flavidulus* from China, just one was shared with Pakistan. The 10 records of *S*. *dorsalis* from Pakistan combined with 239 from GenBank (14 countries) included 99 haplotypes forming five large (>10 sequences) and six small (<3 sequences) clusters (Fig 7). The largest haplotype cluster included three subclusters; the first two subclusters included haplotypes from Asia (China, India, Israel, Japan, Pakistan, Thailand, Vietnam) and Africa (Kenya), while the third included haplotypes from Asia (China, Japan, Singapore, Taiwan, Thailand), and North America (USA). The second and third largest clusters were comprised of haplotypes predominantly from Japan and China. Seven haplotypes of *S*. *dorsalis* were found in Pakistan with three shared with India (Fig 7). The 11 haplotype clusters corresponded to the same number of BINs for this species on BOLD (AAC9747, AAC9749, AAC9750, AAC9751, ACQ0434, ACQ0435, ACQ4218, ACV6509, ACV6510, ACV6511, ACV7644) (Fig 7).

## Discussion

The thrips fauna of Pakistan is poorly known and no molecular data was available before this study which coupled morphological identifications with subsequent barcode analysis. Morphological study resolved most thrips to a presumptive species, but 34 specimens could only be identified to a genus or family. Because thrips are so difficult to identify morphologically, the development of DNA-based identification system is an attractive option [59]. However, the use of molecular approaches requires a reference sequence library from well-identified specimens; its lack inevitably compromises efforts to make taxonomic assignments by molecular analysis [60]. Efforts to build a DNA barcode library for thrips have begun [61], but just 263 of the 6000 known species of Thysanoptera have a barcode record and nearly 10% of these records derive from the present study. Consequently, sequence matches were only found for 13 of the 56 species analyzed in this study and none of these reference sequences derived from Pakistan. This gap highlights the need to further populate databases with reference sequences from carefully identified specimens. This study provides barcode data for 56 lineages of Thysanoptera, 42 identified to the species-level. Aside from providing the first barcode records for Thysanoptera from Pakistan, this study raised the species count for the country from 77 to 87.

Barcode gap analysis showed that the maximum intraspecific distance was invariably lower than the NN distance, even for those species (*A*. *intermedius*, *H*. *reuteri*, *T*. *palmi*, *T*. *tabaci*) with high intraspecific divergence (>2%). Two of these cases (*T*. *palmi*, *T*. *tabaci*) were expected as prior studies [14,31] have reported high intraspecific distances and multiple COI clusters in these species. For example, in a multi-country analysis of COI, Kadirvel et al. [14] found a maximum K2P distance of 19.9% for *T*. *palmi* and 10.4% for *T*. *tabaci* with the former species showing four and the latter three COI variants. In fact, Rebijith [31] reported maximum barcode divergences of 12.3% and 13.8% for *T*. *palmi* and *T*. *tabaci* in Indian populations, much higher values than those detected in these species from Pakistan. We did not perform NJ or phylogenetic analysis with the global data for these species, but we performed distance analysis and constructed MSN of haplotypes. The distance analysis showed a maximum divergence of 13% in *T*. *palmi* and 12% in *T*. *tabaci*, and the MSN showed four haplotype clusters for *T*. *palmi* and three for *T*. *tabaci*. The two haplotype clusters for *T*. *tabaci* were assigned to a single BIN by BOLD, while sequences from the most divergent cluster for this species remain without a BIN because they were <500 bp. Similarly, two main haplotype clusters for *T*. *palmi* were assigned to two BINs, while two additional clusters, each with one sequence (<500 bp) were not assigned to a BIN because they did not meet quality criteria (e.g., >500bp, <1% ambiguous bases), and the sequences without a BIN, in the haplotype network analysis, fall under the short-length category. However, the number of haplotype clusters recognized in our analysis corresponds with the number of NJ clusters reported for *T*. *palmi* and *T*. *tabaci* by Kadirvel et al. [14]. The gap between maximum intraspecific and minimum interspecific distances has been frequently used for species delimitation in various animal groups [54,62,63]. Prevot et al. [64] used barcode gap analysis, with several other species delimitation methods, to identify species in a complex group of snails. Likewise, Roy et al. [65] and Ashfaq et al. [66] employed barcode gap analysis as a tool to discriminate cryptic species of termites and butterflies, respectively. However, other reports have shown the absence of a barcode gap in well-differentiated [67] and recently diverged [68] species.

The present study gathered barcode records from 42 of the 43 morphological species that were collected. Specimens from 39 of these species were assigned to a single BIN, indicating perfect congruence with morphological identifications. The other three species (*A*. *intermedius*, *H*. *reuteri*, *T*. *palmi*) showed BIN splits. The number of BINs likely signals the number of species in our collection as except for *T*. *tabaci* where the only BIN was split into two clusters, similar number of clusters was recovered by both NJ and Bayesian analysis. Researchers have used BINs to delimit species in barcode datasets and have found them congruent with the morphospecies [22].

The barcode sequences from *A*. *intermedius*, *H*. *reuteri*, *T*. *palmi* and *T*. *tabaci* showed deep divergences suggesting that each is a cryptic species complex. This inference was supported by the divergences observed in ITS1. Although inter-BIN ITS1divergence was high in *H*. *reuteri* (4.3%) and *T*. *palmi* (2.3%), it was relatively low in *A*. *intermedius* (1.4%) and *T*. *tabaci* (1.5%). Although the utility of ITS1 for differentiating cryptic species has been documented [69], some taxa show lower divergence in ITS1 than COI [70] limiting the usefulness of this marker in discriminating closely related species. For example, in a group of Leptaxini gastropods where COI revealed high divergence, ITS1 and ITS2 showed little variation [71]. The NJ analysis of COI-3ʹ sequences for *T*. *palmi* and *T*. *tabaci* from Pakistan confirmed that these species matched their respective counterparts in GenBank with each species including at least two genetic clusters, a result supporting the similar divergence observed in barcode sequences from these two species. While earlier morphological studies suggested that *T*. *tabaci* is a complex of several species [72], color and size variation has hampered their discrimination [4]. Similar suggestions for the presence of a species complex have been made for *T*. *palmi* [27] and *S*. *dorsalis* [73,74]. Revelation of cryptic species by barcode data has been documented in other insects including sphingid moths [75], leaf-mining micromoths [76], aphids [77] and whiteflies [78]. However, further studies are needed to confirm the reproductive isolation of these cryptic thrips lineages, as has been done for *F*. *occidentalis* [29].

The present study has indicated that barcode haplotypes for the four vector species from sites around the world were clustered in groups, information that may be useful in analyzing vector-virus relationships and disease epidemiology as virus transmission capacity is known to vary among species populations [79]. Several thrips species, including important virus vectors, are now thought to be species complexes [31], but there is little information on the role of each lineage in viral transmission. For example, a recent study [74] suggests that *S*. *dorsalis* is a complex of at least nine species, but that just one member of this complex, South Asia 1, is involved in virus transmission. In this study, the MSN for *S*. *dorsalis* revealed 11 divergent haplotype clusters corresponding to the 11 BINs for this species on BOLD. If the BINs are species proxies, *S*. *dorsalis* is a complex of 11 cryptic species, two more than reported by Dickey et al. [74]. This result highlights the need to gain an understanding of both the thrips species complexes and their genetic lineages through development of a comprehensive global DNA barcode library for Thysanoptera. This study helps to address this need by employing DNA barcodes to examine thrips diversity in Pakistan.

## Supporting Information

S1 Appendix Checklist of the 87 species of Thysanoptera recorded from Pakistan. Species analyzed in this study are shown in bold.
(DOC)

S1 Table BOLD Process IDs and GenBank accessions from this study and from BOLD/GenBank. (XLS)
