# Supplementary material for: DNA Barcode Analysis of Thrips (Thysanoptera) Diversity in Pakistan Reveals Cryptic Species Complexes
Source: PLoS One. 2016 Jan 7;11(1):e0146014. doi: 10.1371/journal.pone.0146014 (PMC4704811; doi:10.1371/journal.pone.0146014)
Supplement: S1 Appendix — Species analyzed in this study are shown in bold. (DOC) [file pone.0146014.s001.doc]

**Appendix 1:** Checklist of the 87 species of Thysanoptera recorded from Pakistan. Species analyzed in this study are shown in bold.

| **S.No.** | **Genus** | | **Species** | **Reference** |
| --- | --- | --- | --- | --- |
| **A) Family Phlaeothripidae** | | | | |
| 1 | *Bamboosiella* Ananthakrishnan | | *Bamboosiella murreensis* | Saeed & Yousuf 1994 |
| 2 |  | | *Bamboosiella varia*  Ananthakrishnan and Jagadish | Akram 2000 |
| 3 | *Allothrips* Hood | | *Allothrips pillichellus* Priesner | Akram *et al*. 2003b |
| 4 | *Apterygothrips* Priesner | | ***Apterygothrips pellucidus* (Ananthakrishnan) *** | Present study |
| 5 | *Ecacanthothrips* Bagnall | | *Ecacanthothrips tibialis* (Ashmead) | Akram 2000 |
| 6 | *Ethirothrips* Karny | | *Ethirothrips longisetis* (Ananthakrishnan and Jagadish) | Akram *et al*. 2003b |
| 7 | *Gynaikothrips* Zimmermann | | *Gynaikothrips khushabensis* | Saeed & Yousuf 1994 |
| 8 |  | | *Gynaikothrips robustus* | Saeed & Yousuf 1994 |
| 9 | *Haplothrips* Amyot and Serville  subgenus *Haplothrips* | | ***Haplothrips* (H.) *bagrolis* Bhatti** | Ali, 1976 |
| 10 |  | | ***Haplothrips* (H.) *ciliatus*** | Saeed & Yousuf 1994 |
| 11 |  | | ***Haplothrips* (H.) *ganglbaueri* Schmutz** | Ali 1976 |
| 12 |  | | ***Haplothrips* (H.) *gowdeyi* (Franklin)** | Saeed & Yousuf 1994 |
| 13 |  | | *Haplothrips* (H.) *longisetosus* Ananthakrishnan | Saeed & Yousuf 1994 |
| 14 |  | | ***Haplothrips* (H.) *stylatus*** | Saeed & Yousuf 1994 |
| 15 |  | | ***Haplothrips* (H.) *tenuipennis* Bagnall** | Saeed & Yousuf 1994 |
| 16 |  | | ***Haplothrips* (H.) *andresi* Priesner** | Akram 2000 |
| 17 |  | | *Haplothrips* (H.) *bicolor* (Ananthakrishnan) | Akram 2000 |
| 18 |  | | *Haplothrips* (H.) *ceylonicus* Schmutz | Akram 2000 |
| 19 |  | | ***Haplothrips* (H.) *reuteri*  Karny** | Akram 2000 |
| 20 |  | | *Haplothrips* (H.) *howei* (Mound & Minaei, 2007) | Akram 2000 |
| 21 | *Trybomiella* Bagnall (subgenus) | | *Haplothrips* (T.) *clarisetis* Priesner | Saeed & Yousuf 1994 |
| 22 | *Plicothrips*Bhatti | | ***Plicothrips apicalis* Bagnall** | Ali 1976 |
| 23 | *Ananthakrishnana* Bhatti | | ***Ananthakrishnana* *euphorbiae* Priesner** | Saeed & Yousuf 1994 |
| 24 | *Liothrips* Uzel | | *Liothrips aberrans* Muraleedharan and Sen | Akram 2000 |
| 25 |  | | *Liothrips bournieri* Sen | Akram 2000 |
| 26 |  | | ***Liothrips infrequens* Muraleedharan and Sen** | Akram 2000 |
| **B) Family Aeolothripidae** | | | | |
| 27 | *Aeolothrips* Haliday | | *Aeolothrips distinctus* Bhatti | Saeed & Yousuf 1994 |
| 28 |  | | ***Aeolothrips intermedius* Bagnall** | Saeed & Yousuf 1994 |
| 29 |  | | *Aeolothrips collaris* Priesner | Akram 2000 |
| **C) Family Thripidae** | | | | |
| 30 | | *Anaphothrips* Uzel | ***Anaphothrips sudanensis* Trybom** | Akram 2000 |
| 31 | | *Anascirtothrips* Bhatti | *Anascirtothrips arorai* Bhatti | Saeed & Yousuf 1994 |
| 32 | | *Aptinothrips* Haliday | *Aptinothrips rufus* Haliday | Akram 2000 |
| 33 | | *Arorathrips* Bhatti | ***Arorathrips mexicanus* Crawford** | Akram 2000 |
| 34 | | *Astrothrips* Karny | *Astrothrips stannardi* Bhatti | Saeed & Yousuf 1994 |
| 35 | |  | *Astrothrips tumiceps* Karny | Akram 2000 |
| 36 | | *Caliothrips* Daniel | *Caliothrips indicus* Bagnall | Akram 2000 |
| 37 | | *Chaetanaphothrips* Priesner | ***Chaetanaphothrips orchidii* Moulton ***** | Present study |
| 38 | | *Chirothrips* Haliday | *Chirothrips africanus* Priesner | Saeed & Yousuf 1994 |
| 39 | |  | ***Chirothrips meridionalis* Bagnall ***** | Present study |
| 40 | | *Dendrothripoides* Bagnall | *Dendrothripoides ipomoeae* Bagnall | Akram 2000 |
| 41 | |  | ***Dendrothripoides innoxius* ***** | Present study |
| 42 | | *Elbuthrips* Bhatti | *Elbuthrips latis* Bhatti (1973) | Saeed & Yousuf 1994 |
| 43 | | *Fulmekiola* Karny | *Fulmekiola serrata* Kobus | Akram 2000 |
| 44 | | *Frankliniella* Karny | *Frankliniella insularis* Franklin | Saeed & Yousuf 1994 |
| 45 | |  | ***Frankliniella schultzei*** **Trybom** | Ali 1976 |
| 46 | | *Helionothrips* Bagnall | *Helionothrips mube* Kudo | Akram 2000 |
| 47 | | *Hydatothrips* Karny | ***Hydatothrips atactus* Bhatti** | Akram 2000 |
| 48 | |  | *Hydatothrips ekasi* Kudo | Akram 2000 |
| 49 | | *Indothrips* Bhatti | *Indothrips religiosus* | Saeed & Yousuf 1994 |
| 50 | | *Megalurothrips* Bagnall | ***Megalurothrips peculiaris* Bagnall** | Akram 2000 |
| 51 | |  | ***Megalurothrips usitatus* Bagnall ***** | Present study |
| 52 | |  | ***Megalurothrips distalis* Karny ***** | Present study |
| 53 | | *Microcephalothrips* Bagnall | ***Microcephalothrips abdominalis* Crawford** | Ali 1976 |
| 54 | | *Mycterothrips* Trybom | ***Mycterothrips nilgiriensis* Ananthakrishnan** | Akram *et al*. 2002 |
| 55 | | *Bregmatothrips* Hood | [*Bregmatothrips binervis*](http://thrips.info/wiki/Bregmatothrips_binervis)Kobus | Akram 2000 |
| 56 | | *Neohydatothrips* John | ***Neohydatothrips samayunkur* Kudo ***** | Present study |
| 57 | | *Pseudodendrothrips* Schmutz | ***Pseudodendrothrips bhattii* Kudo** | Akram 2000 |
| 58 | | *Rhipiphorothrips* Morgan | *Rhipiphorothrips cruentatus* Hood | Saeed & Yousuf, 1994 |
| 59 | | *Scirtothrips* Shull | *Scirtothrips bispinosus* Bagnall | Saeed *et al*. 1994 |
| 60 | |  | ***Scirtothrips dorsalis* Hood** | Ali 1976 |
| 61 | |  | *Scirtothrips mangiferus* | Saeed *et al*. 1994 |
| 62 | |  | ***Scirtothrips oligochaetus* Karny** | Saeed *et al*. 1994 |
| 63 | | *Scolothrips* Hinds | ***Scolothrips rhagebianus* Priesner** | Saeed & Yousuf 1994 |
| 64 | | *Sorghothrips* Priesner | *Sorghothrips jonnaphilus* Ramakrishna | Saeed & Yousuf 1994 |
| 65 | | *Stenchaetothrips* Bagnall | *Stenchaetothrips biformis* Bagnall | Akram 2000 |
| 66 | |  | *Stenchaetothrips faurei* Bhatti | Akram 2000 |
| 67 | | *Taeniothrips* (Amyot & Serville, 1843) | ***Taeniothrips major* Bagnall***** | Present study |
| 68 | | *Thrips* Linnaeus | ***Thrips alatus* Bhatti** | Akram *et al*. 2003a |
| 69 | |  | ***Thrips apicatus* Priesner** | Saeed & Yousuf 1994 |
| 70 | |  | *Thrips beharensis* Ramakrishna and Margabandhu | Saeed & Yousuf 1994 |
| 71 | |  | ***Thrips carthami* Shumsher** | Palmer 1992 |
| 72 | |  | ***Thrips coloratus* Schmutz** | Palmer 1992 |
| 73 | |  | ***Thrips decens* Palmer** | Akram *et al*. 2003a |
| 74 | |  | *Thrips evulgo* Palmer | Palmer 1992 |
| 75 | |  | *Thrips flavus* Schrank | Palmer 1992 |
| 76 | |  | ***Thrips flavidulus* (Bagnall)** | Palmer 1992 |
| 77 | |  | ***Thrips florum* Schmutz** | Akram *et al*. 2003a |
| 78 | |  | *Thrips garuda* Bhatti | Akram *et al*. 2003a |
| 79 | |  | ***Thrips hawaiiensis* Morgan** | Palmer 1992 |
| 80 | |  | *Thrips kodaikanalensis* Ananthakrishnan and Jagadish | Akram *et al*. 2000 |
| 81 | |  | *Thrips orientalis* Bagnall | Saeed & Yousuf 1994 |
| 82 | |  | ***Thrips palmi* Karny** | Palmer 1992 |
| 83 | |  | *Thrips subnudula* Karny | Palmer 1992 |
| 84 | |  | ***Thrips tabaci* Lindemann** | Palmer 1992 |
| 85 | |  | ***Thrips trehernei* Priesner *** | Present study |
| 86 | |  | *Thrips unonae* Priesner | Akram *et al*., 2003a |
| 87 | | *Lefroyothrips* Priesner | ***Lefroyothrips lefroyi* Bagnall *** | Present study |

(*) First record in Pakistan
